# Treatment of Varicose Veins

**Published:** 1892-06

**Authors:** 


### TREATMENT OF VARICOSE VEINS.

At a meeting of the Edinburgh Medico-Chirurgical Society, Dr. William Taylor read a paper on the treatment of varicose veins.

The treatment he advocated was blistering. He had discovered its good effects accidentally, when treating a case of gout by means of blisters, and had since tried it in many cases with considerable success, and found it especially useful in old people. He considered blistering to be "eminently restorative" in varicose veins. Dr. Taylor did not believe in elastic stockings, and considered operative treatment unscientific. He supposed that the coats of veins participated in some way, which he could not explain, in the restored vitality set up by the action of the blisters. He found that the veins remained sound for several years, but were then apt to become extended again, and required the treatment to be renewed. Its great advantage was that it could be used in cases where palliative treatment was contra-indicated, and that it always did good to the solid œdema so often associated with the varicose condition.

The details of the treatment were as follows: 1. Remove the cause. 2. Obviate the tendency. 3. Elevate the limb for twenty-four hours. 4. Blister from the foot upward, six inches daily, watching the kidneys and bladder. First paint the part with Rubini's tincture of camphor, then with blistering fluid, and lastly with collodion. The blisters must rise and serum be withdrawn to do good. 5. When whole limb is blistered, apply plaster to the whole length of the vein for two weeks. 6. Remove the plaster and see what is the result of the treatment. If the veins bulge, blister again; if they are all right, apply more plaster and allow gentle exercise for a week. If at the end of that time the veins ase still in good condition no further treatment is needed.—*Provincial Med. Journal.*

